# Bioprinting of 3D Adipose Tissue Models Using a GelMA-Bioink with Human Mature Adipocytes or Human Adipose-Derived Stem Cells

**DOI:** 10.3390/gels8100611

**Published:** 2022-09-25

**Authors:** Franziska B. Albrecht, Freia F. Schmidt, Ann-Cathrin Volz, Petra J. Kluger

**Affiliations:** 1 Reutlingen Research Institute, Reutlingen University, 72762 Reutlingen, Germany; 2 Faculty of Natural Science, University of Hohenheim, 70599 Stuttgart, Germany; 3 Faculty of Applied Chemistry, Reutlingen University, 72762 Reutlingen, Germany

**Keywords:** adipose tissue, 3D bioprinting, hydrogels, primary human adipose-derived stem cells, primary human mature adipocytes, gelatin methacryloyl

## Abstract

Adipose tissue is related to the development and manifestation of multiple diseases, demonstrating the importance of suitable in vitro models for research purposes. In this study, adipose tissue lobuli were explanted, cultured, and used as an adipose tissue control to evaluate in vitro generated adipose tissue models. During culture, lobule exhibited a stable weight, lactate dehydrogenase, and glycerol release over 15 days. For building up in vitro adipose tissue models, we adapted the biomaterial gelatin methacryloyl (GelMA) composition and handling to homogeneously mix and bioprint human primary mature adipocytes (MA) and adipose-derived stem cells (ASCs), respectively. Accelerated cooling of the bioink turned out to be essential for the homogeneous distribution of lipid-filled MAs in the hydrogel. Last, we compared manual and bioprinted GelMA hydrogels with MA or ASCs and the explanted lobules to evaluate the impact of the printing process and rate the models concerning the physiological reference. The viability analyses demonstrated no significant difference between the groups due to additive manufacturing. The staining of intracellular lipids and perilipin A suggest that GelMA is well suited for ASCs and MA. Therefore, we successfully constructed physiological in vitro models by bioprinting MA-containing GelMA bioinks.

## 1. Introduction

Various adipose tissue pathologies and defects address the need for suitable models for clinical applications and research aspects. Adipose tissue engineering offers the possibility to develop bioactive tissue constructs that can mimic adipose tissue in both structure and function. Understanding the complex physiology of this tissue in vivo is of great importance for developing such models. Furthermore, their comparability to the native state is of great interest for interpreting the results obtained in vitro.

Native adipose tissue is a dynamic and multifunctional organ that serves as an energy reservoir, heat insulator, and mechanically protects inner organs [1]. Furthermore, it regulates systemic metabolism and inflammation by secretion of adipokines and is, therefore, endocrine active [2,3]. As a subtype of connective tissue, adipose tissue is organized in lobule. Herein, extracellular matrix (ECM) fibers span a network combining multiple mature adipocytes (MA) with stem cells, premature adipocytes, immune cells, and vascular cells [4]. MAs are the most common and characteristic cell type of adipose tissue [5]. Adipose-derived stem cells (ASCs) represent an important source of multipotent stem cells that could differentiate over premature adipocytes into MAs through adipogenesis [6]. This multi-stage process is induced by activated adipogenic transcription factors and cell cycle proteins. During differentiation, cells lose their multipotency, accumulate lipids, change their morphology and develop insulin sensitivity [3]. In contrast, MAs are terminally differentiated and have a roundish cell morphology with one large lipid-filled vacuole, which takes up to 90% of the cell’s volume. Due to the characteristic vacuole, all cell organelles and the nucleus are localized next to the cell wall [7,8]. The lipogenesis (setup), maintenance, and lipolysis (cleavage) of triacylglycerides occur within (pre)mature adipocytes vacuole [9,10].

The ECM is a cell-promoting environment and defines features such as stiffness, elasticity, and geometry of tissues, so also for adipose tissue, where the matrix of collagen (e.g., Ⅰ, Ⅲ, Ⅳ, VI [11,12]) is spun around the cells as a fine network. Especially during adipogenesis or disease development, this highly dynamic network undergoes structural changes [12,13]. Based on the ECM composition and pre- or absence of different molecules, cell shape, proliferation, migration, differentiation, or tissue development are influenced [14,15]. Whereby collagens ensure structural and mechanical stability, and components such as laminin or fibronectin support cell-matrix adhesion by connecting to integrins or the basal lamina [16]. This demonstrates that the cell-surrounding matrix plays an essential role in tissue function and is, therefore, a crucial component in the construction of in vitro models.

As tissue engineering aims to build tissues as close to in vivo situation as possible, it combines cells, materials, and signals in vitro. In recent years, a lot of work has been carried out to introduce the 3rd dimension into such in vitro models, as two-dimensional (2D) cell cultures lack a complex microenvironment and animal models are highly complex and ethically controversial, three-dimensional (3D) models represent a good alternative for preclinical research, e.g., drug screening.

Multiple polymers are available for the generation of hydrogels to simulate a cell microenvironment in 3D in vitro adipose tissue, e.g., poly-lactide-caprolactone, polysaccharides, cellulose-based materials, collagens, or hyaluronic acid [17,18,19,20]. One frequently used material is gelatin methacryloyl (GelMA). For this material, methacrylic side chains are incorporated into a gelatin molecule by a methacrylic anhydride-mediated reaction [21]. Due to these side groups and in the presence of a photoinitiator, covalent crosslinking via radical polymerization under ultraviolet (UV) light is possible by keeping the gelatin molecule properties. According to the number of methacrylic groups within the molecule (degree of functionalization) and the strength of crosslinking, the resulting hydrogels can be tailored regarding stiffness, pore size, and stability [22]. GelMA combines the advantages of natural and synthetic hydrogels. On the one hand, it naturally includes the peptide motif arginine-glycine-aspartic and matrix metallopeptidases sensitive sequences. On the other hand, GelMA can be adapted to a wide range of applications by adapting the synthesis process, adding additives, and crosslinking [23]. Due to these features, GelMA is a widely used material in bioprinting [24]. The additive setup of in vitro adipose tissue models is gaining more and more importance. Benefits are, among automatization, higher reproducibility and standardization, scale-up possibilities, and the generation of an infinite number of geometries. For biological purposes, extrusion-based bioprinting is the favored technique [25]. Herein a continuous strand of material is extruded and placed layer-by-layer onto a printing surface, creating the desired 3D geometry.

The excellent properties of GelMA in terms of cell compatibility and the previous successful applications of this bioink with many different cell types, e.g., neural stem cells [26,27], blood vessel forming cells [28,29], or muscle cells [30,31], make this material an ideal choice for bioprinting soft tissue, including adipose tissue. Multiple publications on ASCs in combination with GelMA and adipogenic differentiation are available [32,33,34,35]. Successfully bioprinted models with differentiated, viable, and functional cells could already be achieved. However, only a few studies deal with the use of human MAs [36,37]. They are readily available in the human system and can be used directly without the need for a preceding in vitro differentiation, which requires time and materials. They also reflect the ideal physiological phenotype [38]. Nevertheless, their handling still represents a challenge, which is why there is relatively little literature to be found—especially in combination with bioprinting.

To mimic the in vivo-like behavior of adipose tissue as close as possible, we compared the bioprinted GelMA-based cellular models using MAs or adipogenically differentiated ASCs (diffASCs) with manually produced ones and additionally compared them to explanted, cultured adipose tissue lobule as native control.

### The Use of GelMA in Adipose Tissue Engineering

**Figure 1:**
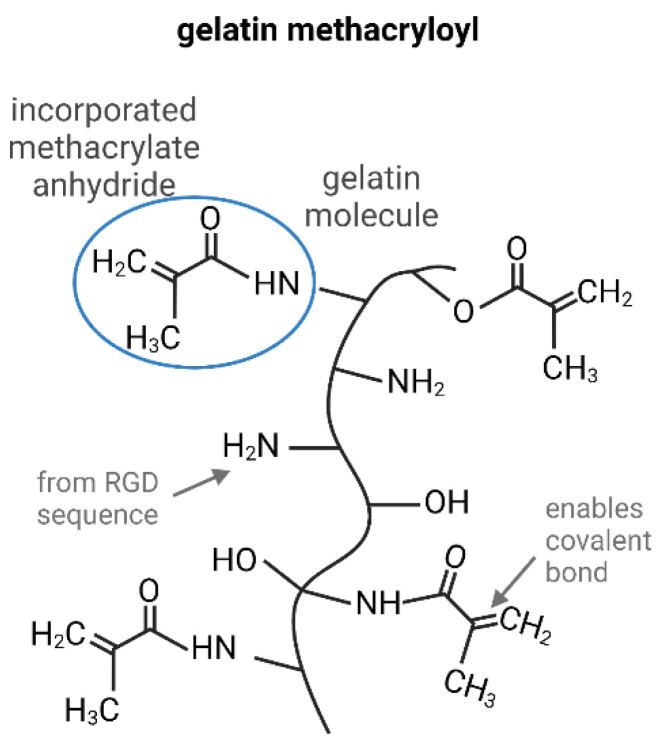
Productions of GelMA with modification sides, inspired by [39].

GelMA is a very commonly used material in tissue engineering, therefore, the production and adaptation of the material characteristics are well-known. Figure 1 shows the GelMA, whereby the side of action within the gelatin molecule is based on the naturally occurring RGD sequence. Due to the addition of methacrylic anhydrides, methyl groups are bound to the nitrogen residue of gelatin. These inserted groups allow covalent crosslinking by radical polymerization in the presence of a photoinitiator.

There are reviews encompassing the use of GelMA for soft tissue engineering, focusing on bioprinting [24] or the adaption of GelMA by material modification [39]. Nevertheless, the summarized literature mainly focuses on wound healing applications, whereby the subdermal layer is not within focus. Therefore, we have compiled a literature excerpt in Table 1 where GelMA was used to construct adipose tissue manually or additively. The hydrogels used have a low weight percent range of GelMA in common and no or only soft additives included. Remarkably, almost all studies relied on the use of ASCs instead of MAs. The literature research revealed that bioprinting (extrusion-based bioprinting, EBB) with GelMA and adipogenic differentiation is rare. No studies could be found on bioprinting MAs in GelMA. This demonstrates that the generation of adipose tissue—particularly through bioprinting and the use of mature cells—can be focused even more strongly.

## 2. Results and Discussion

### 2.1. Explanted Adipose Tissue Lobules Are a Suitable Control for In Vitro Adipose Models

**Figure 2:**
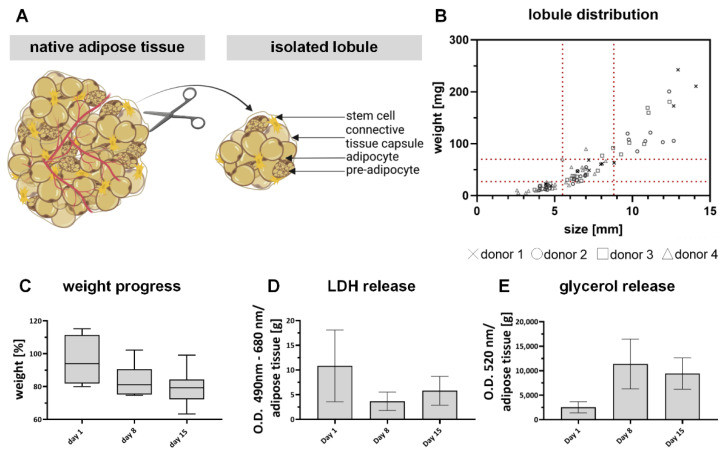
Establishment of lobule culture: (**A**) schematic illustration of lobule isolation and structure; (**B**) distribution of isolated lobules from 4 donors sorted by size-weight ratio. The selected ratio for further trials is shown in between the red lines (**C**) Weight progress of explanted lobules over 15 days. (**D**) Normalized LDH absorbance per gram tissue over 15 days. (**E**) Normalized glycerol release per gram tissue over 15 days.

A positive control based on cultured lobule explants was established to assess the in vivo behavior of the GelMA-based models. For this purpose, intact lobules were obtained from several biological individuals, sorted according to their size and weight, and then examined for stability. The results are summarized in Figure 2. As shown in Figure 2A, multiple lined-up lobules build up the adipose tissue. They represent a small unit in adipose tissue separated by a connective tissue capsule. Different cell types, such as MAs, premature adipocytes, and stem cells, are represented within a lobule [41]. Figure 2B depicts the distribution of isolated lobules from four biological donors, sorted by size-weight ratio. It shows that the variance of the individual lobules within a donor and in adipose tissue can be very wide-ranging, which is a well-known phenomenon described in literature [42]. It might be explained by the natural ability of adipocytes to increase their volume in response to nutrient intake, even up to pathophysiological sizes known as hypertrophy [43,44]. Due to the increased volume of the individual cells, the total volume of the lobule also increases. A representative area with mean size and weight values was defined (within the red markers) to achieve comparable size-weight ratios across biological individuals.

All lobules within this area were examined regarding weight progression (Figure 2C), lactate dehydrogenase (LDH) secretion (Figure 2D), and glycerol release (Figure 2E) throughout the culture period of 15 days. The weight progression shows no significant decrease until day 15 of culture to an average weight of 78 ± 9.4%. The slight and steady reduction in weight is also seen by implanted adipose tissue [45,46] is probably due to the release of cleaved lipids and washed-out cellular debris after cell death. This assumption is supported by the released LDH, as free LDH can be correlated with cell death [47]. Obviously, the largest amount per gram tissue (10.8 ± 6.6 optical density/g tissue) is released on day 1 and thus 24 h after isolation. Until day 8, the released LDH amount decreases and increases again until day 15. The progression suggests that some of the cells directly on the surface were damaged during isolation and released LDH within the first 24 h. The fact that the release decreases until day 8 suggests that the culture is stable and that the culture itself is less stressful than the isolation. The slight increase at the end of culture could indicate that more cells are in an apoptotic state again, which is probably related to the size of the individual lobule. Others have also shown that adipose tissue explants are only stable and, therefore, functional for a limited time [48] but can be used as a control model [49]. The increased glycerol release after 8 days of culture (11,379.4 ± 4704 ng/g tissue) indicates that there might be an increased cleavage of lipids but also a high lipid turnover in general. In summary, we have established a native positive control that can be cultured along with in vitro adipose tissue models and, therefore, can be used to classify the in vivo similarity of the generated models.

### 2.2. Morphological Differences of ASCs and MAs Compared to Native Tissue

**Figure 3:**
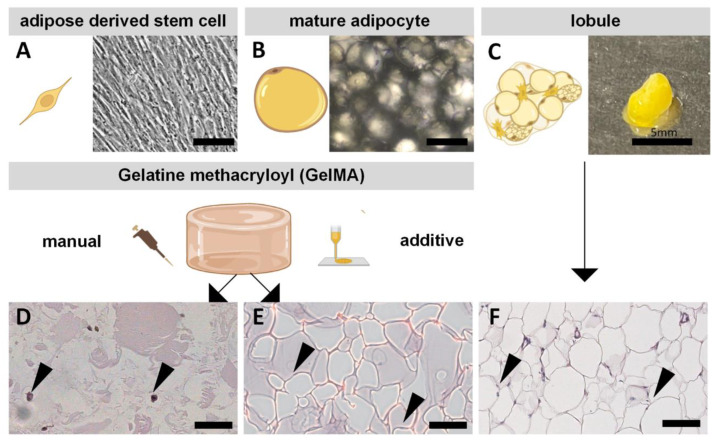
Overview of ASCs and adipocyte morphology in GelMA-based in vitro models and native adipose tissue: schematic illustration and light microscopic images of (**A**) ASCs on tissue culture polystyrene and (**B**) mature adipocytes in suspension culture. (**C**) schematic illustration of a lobule and macroscopic image. Hematoxylin-eosin staining of manually produced ASCs in GelMA on day 15 (**D**), mature adipocytes in GelMA on day 1 (**E**), and explanted lobule on day 1. (**F**) Hematoxylin stains all acidic structures in blue (cell nuclei, indicated by an arrow), eosin stains all alkaline structures in red (mainly GelMA and collagen), and accumulated lipids washed out, visible as white holes; scale bar 100 µm.

Human primary ASCs and MAs were used to construct adipose tissue models (Figure 3). The comparability of the in vitro models to the in vivo functional subunit of adipose tissue, the so-called lobule—was to be shown.

ASCs as precursors of MAs can be differentiated along the mesodermal germ layer [50]—and thus also in the adipogenic direction, while MAs are terminally differentiated cells. The cell types differ morphologically and need different culture conditions. While ASCs are adherent and spindle-shaped (Figure 3A), MAs are large, round, non-adherent cells cultured in suspension (Figure 3B). Both cells are located in adipose tissue and can therefore be isolated from it, as shown in Figure 3C.

For the setup of adipose tissue models, the cells were encapsulated into GelMA and manually or additively set up. In the models with encapsulated ASCs, adipogenic differentiation occurred over two weeks. MA-containing GelMA hydrogels were maintained for one week, the same as the lobuli. The resulting morphology of both manually manufactured models and the lobule was determined by histological hematoxylin-eosin staining and is depicted in Figure 3D–F. As the cell density in native adipose tissue is very high, the matrix content is, therefore, very low. In native tissue, the lipid vacuoles of the MAs appear as roundish white structures in the staining and represent the main component of the tissue. The cells are connected by a small amount of ECM stained in red by eosin. Considering Figure 3D,E, it is recognizable that the GelMA models have fewer white parts and more eosin-stained regions (in this case, GelMA) than the lobule due to the lower cell-matrix ratio of the models. This shows that diffASCs are not yet comparable to the native state, but mature adipocytes are. In general, the cell density within the model cannot be increased arbitrarily since only a certain number/amount of cells can be homogeneously introduced into the GelMA matrix. Otherwise, the matrix amount is too small to provide a stable network. However, in the models with MAs, the characteristic big structures of the lipid vacuoles are clearly visible and thus have a stronger histological resemblance to the native tissue. Even after two weeks of differentiation, diffASCs still have a relatively low lipid content compared to MAs within GelMA, as only small white spots around the dark nucleus are visible (Figure 3D). It is already becoming apparent that, from a morphological point of view, MAs are more similar to native tissue than diffASCs.

### 2.3. Altering GelMA Composition and Bioprinting Parameters Have Stronger Effects on MAs Than on ASCs

**Figure 4:**
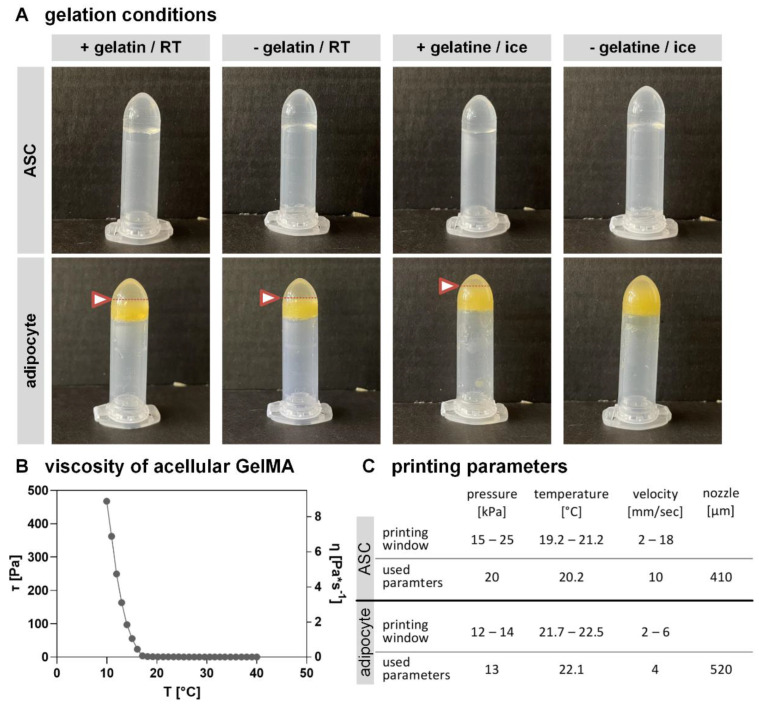
GelMA characteristics and gelation behavior and bioprinting parameters of cell-containing GelMA. (**A**) depicts GelMA containing ASCs or mature adipocytes with (+) and without (−) unmodified gelatin with induced gelation at room temperature (RT) or on ice. Phase-separation indicated by the arrow with dotted line (**B**) Viscosity of acellular GelMA without unmodified gelatin (**C**) listed bioprinting windows and used parameters for ASC or adipocyte-containing GelMA bioink.

As stated above, ASCs and MAs exhibit very distinct characteristics in culture. Therefore, the bioink handling and composition were individually adjusted to each cell type to achieve a homogenous bioink and prevent cell damage during the model setup. The adaptation of the gelation conditions of the GelMA bioink for the respective cell types is shown in Figure 4A. For some parameters (GM2/ gelatin concentration, crosslinking), it was possible to use other studies [36,51] as a reference point. Unmodified gelatin was added to gain a more viscous bioink and to increase the print fidelity [52], as, at room temperature (RT), the storage modulus dominates the loss modulus. Further, the gelation temperature was varied to achieve an accelerated cool down, initializing the well-known temperature-dependent gelation of gelatin [53]. In the upper row, ASC-containing GelMA is depicted with (+) and without (−) additional unmodified gelatin, gelled at RT and on ice. It is evident that all combinations lead to a homogenously gelled bioink without any phase separation. ASCs, as small and adherent cells, can easily be mixed into the water-based GelMA solution, resulting in a stable and uniform bioink with each gelation condition and added gelatin amount used (Figure 4A).

The lower row shows GelMA with incorporated MAs under the same conditions. In contrast to ASCs, 3 out of 4 adipocyte-containing GelMA variations resulted in phase separation. This can be observed by the fact that there is a yellow cellular layer in the lower area and a whitish layer above it (boundary indicated by an arrow and dotted line). This phase separation of lipid-containing cells and gelled water-soluble components is caused by the lower density of mature adipocytes (density of adipose tissue 0.9–0.97 g/cm^3^ [54]) than that of water. The density-based separation happened faster than the temperature-based gelation of the modified and unmodified gelatin molecules. Therefore, a homogeneously gelled bioink for MAs was only obtained on ice with a fast gelation process and without adding unmodified gelatin, as the accelerated cool down prevented the density-based cell separation. The fact that accelerated cooling on ice with unmodified gelatin did not result in a completely homogeneous bioink could be related to the resulting network size. As a higher gelatin concentration within a solution leads to a denser network [55], it might be the case that some bigger adipocytes could not be mixed into the solution and therefore show a slight phase separation.

Further, the water uptake is decreased with increasing gelatin concentration [55], which might lead to stiffer hydrogels that are not favored for adipose tissue [56]. After the adaption of an accelerated cool-down on ice, a stable, homogenously mixed bioink was achieved for MAs and ASCs. Within Figure 4B the viscosity of the used cell-free GelMA solution without any unmodified gelatin is depicted. It is evident that the GelMA exhibits temperature-dependent properties as the shear stress drops with increasing temperature. Similar results are observed for the viscosity, which decreases with rising temperature. The used GelMA shows a higher viscosity under 17 °C, which decreases above this temperature. In general, the material offers a suitable behavior for bioprinting soft tissue engineering, especially for adipose tissue.

As MAs and ASCs have very different cell sizes and morphologies, also the bioprinting process was individually adapted for each cell type. The parameters temperature, pressure, and velocity were systematically varied as they all impact cell viability and printing outcome [57]. As recommended in literature [58], a usable printing window for ASC- or MA-containing GelMA was defined (provided as supplement S1), and afterward, the parameters that allow bioprinting with high cell viability and sufficient print fidelity were defined (Figure 4C). The ASC-containing bioink can be used in a broad range of bioprinting conditions. In contrast, only a small bioprinting window leads to acceptable models containing MAs. For both cell-containing bioinks, values out of the window lead to randomly extruded patterns. While high temperatures and low pressure gained randomly distributed dots, increased pressure and decreased temperature gained insufficiently printed or half-sided models. In contrast, higher temperatures and higher pressure have caused an undefinable bulk of the material. By adjusting the velocity, the phenomena described could be somewhat mitigated but not diminished.

The differences in the set parameters for ASCs and MAs can be explained by the different biological behavior of the cell types. While it is widely spread to bioprint ASCs [59], no studies could be found on bioprinting encapsulated MA-hydrogels. Focusing on MAs, their sensitive reaction to shear stress might explain the need for lower pressure and a slower velocity during the bioprinting process compared to ASCs. MAs react sensitive to deformation and exhibit an elliptical shape depending on compression [60,61]. Slower velocity and reduced pressure led to less shear stress and, thus, in milder process conditions, which are highly important for MAs. Further, the different morphology of the cell types might explain the higher temperature needed for bioprinting MAs. It might result from the fact that they make up a larger part of the volume within the bioink than small adherent cells such as ASCs. The lipids within the vacuoles need higher temperatures to reach the same viscosity [62] as the GelMA solution already has at lower ones. Finally, a larger nozzle diameter is required due to the large cell diameter of mature adipocytes and due to their higher vulnerability against mechanical cues. In general, it can be concluded that the establishment of the GelMA bioink and the bioprinting process for MAs was significantly more difficult and time-consuming than for ASCs. Nevertheless, this step is recommended, as MAs represent the mature and characteristic cell type of adipose tissue and thus also a good candidate for the construction of in vivo-close tissue models.

### 2.4. Both ASC and MAs in GelMA Exhibit High Cell Viability after Manual and Additive Setup Comparable to Explanted Lobule

After establishing an in vitro model control, gaining a homogenous bioink, and evaluating the bioprinting window with final parameters, cell-containing GelMA was manually and additively set up and compared with an explanted lobule regarding viability. This comparison aimed to show the influences caused by the bioprinting process as it can influence cell viability due to enhanced pressure during extrusion and long fabrication times [63].

**Figure 5:**
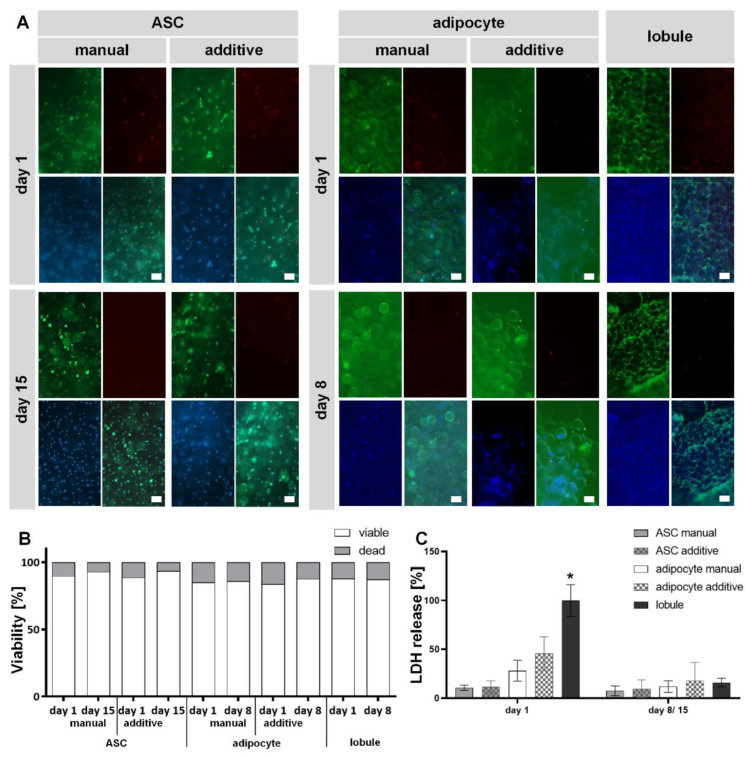
Viability analysis of encapsulated diffASCs and mature adipocytes in GelMA compared to explanted adipose tissue. (**A**) Live-dead staining (viable cells in green by FDA, dead cells in red by propidium iodide, cell nuclei in blue by Hoechst) of manual and bioprinted GelMA hydrogels containing ASCs or mature adipocytes on days 1 and 8 or 15, respectively, compared to lobules on days 1 and 8. Top left FDA, top right PI, bottom left Hoechst, and bottom right merged. Scale bar 100 µM (**B**) semi-quantitative viability analysis of counted viable and dead cells per condition. N = 9 (**C**) normalized LDH release of manual and additively manufactured GelMA hydrogels containing ASCs or mature adipocytes, respectively, relative to lobules. * α ≤ 0.05.

Live-dead staining with fluorescein diacetate (FDA) and propidium iodide (PI) for ASCs on days 1 and 15 and for MAs and lobule on days 1 and 8 are shown in Figure 5A and the semi-quantification of the images in Figure 5B. Viable cells are depicted in green by FDA (only the cytoplasm), dead ones in red by PI (only the cell nuclei), and the cell nuclei are counterstained with Hoechst in blue. Only a few dead cells are detected on day 1 for all manual and additive cell-containing hydrogels. All visible cells are viable at other time points and in the explanted lobule. Further, there is no obviously noticeable difference concerning the method of manufacture. Since only a few dead cells can be seen in all models 24 h after setup, it can be assumed that the process conditions are only minimally harmful to the cells. Furthermore, there are no differences between the manually and additively produced models (neither for ASCs nor for MAs), indicating a well-established and mild bioprinting process. The semi-quantification of the cell viability of all models, supports this assumption. MAs show slightly reduced viability compared to ASCs, which increases again somewhat with continued culture (individual values are available as supplement S2). The fact that ASCs can be encapsulated in GelMA and exhibit high viability after bioprinting is also demonstrated by literature—although more frequently with subsequent differentiation in other directions [24]. For MAs, there is little comparable literature so far. Nevertheless, Huber et al. have previously demonstrated that GelMA is a suitable material for encapsulation of MAs [36]. Louis et al. have succeeded in bioprinting MAs in collagen with endothelial cells and demonstrated cellular viability and function after seven days [37]. The explanted lobules also showed high viability on both days. Nevertheless, we do not assume that the possibly isolated blood vessels significantly influence the supply. Since both for preserving ex vivo blood vessels [64] and organs [65], a perfusion culture is helpful and useful for maintaining functionality.

The results from the live-dead staining were additionally supported by determining the release of LDH, which is associated with the destruction of cell membrane integrity and, thus, with cell death. Figure 5C shows the LDH release of all hydrogel variants normalized to the explanted lobule on day 1. It turns out that the lobule control on day 1 has a significantly higher secretion of LDH. As expected, the explanted lobule showed the highest LDH level on day 1. It is likely because the mechanical stress on the cells is greatest during isolation and the risk of injuring them is very high. Furthermore, it can be assumed that the lobuli contain the largest number of cells that can potentially release LDH.

In general, three trends can be observed: hydrogels with MAs have a higher secretion than those with stem cells, additively manufactured models have an increased secretion compared to manual ones, and the secretion decreases from time point 1 to time point 2 for all hydrogel variants and lobules. Since MAs are damaged at strains over 30% [66], it is a possible explanation for the higher LDH release by MAs compared to ASCs. Both cell types show a higher LDH release after being processed by additive manufacturing. This might be explained by more cells being exposed to the shear stress during the filament extrusion than by manual pipetting. Not to be neglected is that LDH secretion decreases over time for all models. This suggests that the cells have recovered after the setup and are still viable, confirmed by live-dead staining on days 15 or 8, respectively. Therefore, measured LDH release and detected cell death occurred independently from the manufacturing method and can be attributed to other circumstances such as physiological cell cycles.

In summary, a comparatively mild bioprinting process for ASCs and MAs could be established, in which the encapsulated cells show high viability.

### 2.5. ASCs Successfully Differentiated Adipogenically, and MAs Maintained Their Function in Bioprinted and Non-Printed GelMA Models

**Figure 6:**
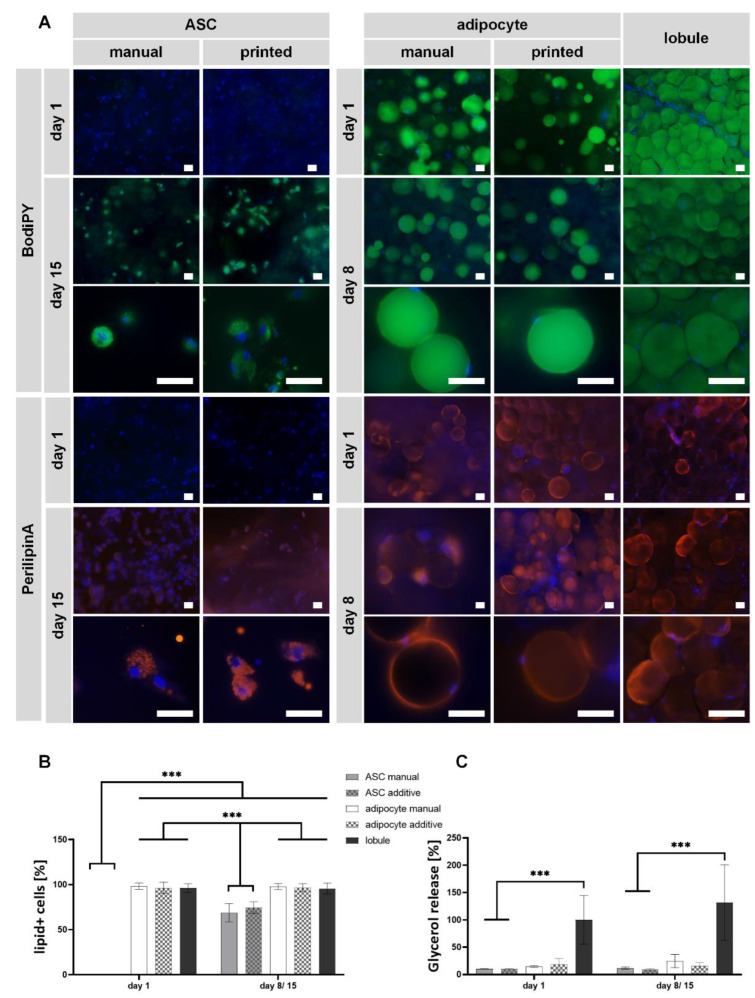
Functionality staining of encapsulated and bioprinted cells compared to lobules. (**A**) Staining of intracellular lipids (in green by BodiPY) and cell nuclei (in blue by Hoechst) as well as perilipin A (in orange by anti perilipin A antibody) and cell nuclei (in blue by DAPI) of manual and bioprinted GelMA hydrogels with encapsulated ASCs on days 1 and 15, encapsulated mature adipocytes on days 1 and 8, and explanted lobules on days 1 and 8. Scale bar 100 µM. (**B**) semi-quantitative determination of lipid-positive cells (**C**) Normalized glycerol release of ASCs, mature adipocytes, and native adipose tissue on days 1 and 8 or 15, respectively, using manual or additive setups. *** α ≤ 0.001.

To finally evaluate the models’ functionality, the characteristics of diffASCs and MAs in GelMA were compared to the explanted lobules. The characteristic markers used for this purpose were the staining of intracellular lipids and protective lipid-membrane protein perilipin A, shown in Figure 6A. While stored lipids indicate the cellular state, perilipin A prevents the intracellular lipids from degradation through lipases.

As expected, intracellular lipids (shown in green) in ASCs are not present at day 1—neither for bioprinted nor for manual models. Until day 14, intracellular lipids were generated, and differentiated ASCs exhibited a more roundish cell morphology with multiple small vacuoles around the cell nuclei in both model setup types. The same phenomena are also observed for perilipin A staining. Here, the protective layer of the lipid vacuole (shown in orange) covers the whole small vacuole on day 14. In general, the staining displayed for ASCs in printed and non-printed models seems to be non-different. The absence of intracellular lipids and perilipin A on day 1 is in line with expectations because there were no stimuli for ASC to differentiate at that time. It was also expected that ASCs at day 15 have multiple, small lipid vacuoles containing lipids surrounded by perilipin A as adipogenic differentiation potential was previously demonstrated in GelMA [32,33,34,35]. Summarized, the manufacturing method shows no effect on the adipogenic differentiation potential within GelMA hydrogels.

MAs in GelMA hydrogels exhibit an univacuolar morphology on days 1 and 8, as shown in both manufacturing methods’ stainings. The cell nucleus (blue) is placed directly next to the lipid-filled vacuole (green). During culture, no optical changes in the cells are recognizable, indicating a stable cell state of MAs in GelMA. No signs of dedifferentiation (lipolysis) are visible, which others have also been able to show by culturing MAs under membranes [67] or within chip systems [68,69] as other possibilities to culture viable MAs. The same behavior is also seen for the perilipin A staining. Here, the cells express a continuous perilipin A layer (orange) on the lipid vacuole on both days. All these observations are identical for additive and manual models, as no significant differences can be found. It was expected that MAs have an univacuolar morphology, thus, the main part of the cells is lipid-filled. Similar holds for the expression of perilipin A, the continuous ring around the large lipid vacuole corresponds to the expectations. The explanted lobule shows an univacuolar morphology for all cells, which is not always round due to the high cell density. Overall, the same behavior can be observed for MAs in GelMA. Comparing the hydrogels to native adipose tissue, it is evident that MAs exhibit a morphology closer to the native state than differentiated ASCs. This is shown by the total size of the cells as well as by the size and number of the individual vacuoles. Nevertheless, it may also be possible to mature ASCs into a univacuolar morphology, as we have shown for another material [19].

The images were semi-quantified by counting lipid-positive (lipid+) cells at each time point to support the results (Figure 6B). While almost 100% of the cells in the AC-containing hydrogels and lobules show accumulated lipids, significantly fewer cells in the ASC hydrogels have lipids. This holds for day 1 and day 15, even if the increase from day 1 to day 15 is significant. There is no visible or significant difference between the manufacturing methods.

In addition to the staining, the release of glycerol was determined (Figure 6C). Higher glycerol levels indicate lipid cleavage during dedifferentiation, but there is a correlation between the amount of glycerol and general lipid turnover [70]. All values are normalized to the glycerol release of lobules on day 1. ASC-containing hydrogels (bioprinted or not) have a significantly lower amount of released glycerol than lobules on days 1 (10.4% ± 0.4% manual; 10.5% ± 0.4% additive) and 15 (11.8% ± 1.9% manual; 9.3% ± 1.5% additive). Glycerol release increases only slightly during differentiation, again indicating cells are not in a fully matured state. For adipocyte-containing hydrogels, the secretion is not significantly lower than that of lobules on days 1 (14.6% ± 1.6% manual; 18.8% ± 10.5% additive) and 8 (24.6% ± 12.0% manual; 16.2% ± 5.8% additive). As MAs in GelMA are able to preserve the glycerol release, it can be concluded that the cells maintain their function, which in turn is supported by lipid staining. The slightly higher glycerol diffusion of the manual models on day 8 might appear due to the lower nutrient diffusion compared to the additive models.

Summarized, functionality analysis underlines the observations that the bioprinting process does not significantly influence the encapsulated cells. All cells stored or maintained intracellular lipids and expressed the characteristic protein perilipin A. The manufacturing process for models generated with MAs or ASCs did not affect the lipolysis rate measured by a glycerol assay. Nevertheless, as previously shown [38], MAs generally mimic the native cell state of adipose tissue more closely than diffASCs. This observation can also be supported by our results, which is a reason why it is advisable to use MAs for in vivo close adipose tissue models instead of ASCs.

## 3. Conclusions

In this study, we demonstrated that GelMA, a suitable biomaterial for 3D bioprinting of human primary ASCs, can also be used for bioprinting mature adipocytes—what we have shown for the first time. Further, we established a lobule explant culture as a native control to make a statement about the models in terms of their in vivo similarity. Compared to the manual setup, the bioprinting process described herein had no harmful effects on the adipogenic differentiation potential or lipid maintenance of the encapsulated cells. All encapsulated cells stored (ASCs) or maintained (MAs) intracellular lipids and expressed perilipin A, indicating their adipocyte phenotype and function. Comparing the two cell types in GelMA with the explanted lobules shows that MAs in GelMA resemble explanted lobules—and therefore the native state—more closely than stem cells regarding morphology, secretome, and function. The results shown here prove that it is possible to bioprint viable and functional MAs in GelMA, which should also be considered for other studies to further increase the similarity of in vitro models to the native state.

## 4. Materials and Methods

### 4.1. Lobule Isolation and Culture

Used lobule, primary human mature adipocytes (MAs), and human adipose-derived stem cells (ASCs) were isolated from adult subcutaneous tissue samples from elective surgeries.

To dissect intact lobules, small pieces of subcutaneous adipose tissue with little connective tissue were carefully cut off using forceps and tissue scissors. Next, the individual lobules were carefully separated from these pieces by cutting along the connective tissue around the individual cell clusters. Most lobules exhibited a roundish to oval shape with no discernible edges.

Lobules were cultured in 750 µL Adipocyte Maintenance 1 PRF (AM1, amsbio, Abington, U.K.) supplemented with 1% Penicillin/ Streptomycin (P/S, Biozym, Hessisch Oldendorf, Germany) with twice-weekly media exchanges.

*Weight Size and Size Measurement*

Lobuli weight was determined after carefully removing excess liquid from the surface with sterilized lint-free wipes (Kimtech, Kimberly-Clark, Oldendorf, Germany) and weighed. The weight progress was calculated with the following Formula (1):

(1) $$\frac{100\times sample\;weight}{initial\;weight}$$

The volume was calculated by measuring the height and the diameter with a digital caliper (Alpha Tools, Mannheim, Germany) via the following Formula (2):

(2) $$2\times \pi \times height\times \frac{diameter}{2}$$

### 4.2. Cell Isolation

Cells were isolated according to previously published protocols [36,71]. Briefly, the adipose layer was cut into small pieces and digested in 100 U/mL collagenase (Serva Electrophoresis, Heidelberg, Germany) solution in Dulbecco’s Modified Eagle Medium (DMEM high glucose, Pan-Biotech, Aidenbach, Germany) with 1% bovine serum albumin (Biomol, Hamburg, Germany). Digestion took place for 1.5 h at 37 °C under mild agitation. The resulting solution was strained twice through a 500 µm and afterward through a 200 µm strainer. Within 10 min, the filtrate settled in an upper layer liquid, where the mature adipocytes were collected, and a lower liquid layer, including remaining tissue residues. The lower layer was further digested for 1.5 h to isolate ASCs. Next, mature adipocytes were washed twice using DMEM at room temperature (RT) for 10 min and once with DMEM/Ham’s F-12 (Pan-Biotech) supplemented with 3% fetal calf serum (Thermo Fisher Scientific, MA, USA) at 37 °C, 5% CO_2_ for 1 h. After a final washing step with DMEM (10 min at RT), mature adipocytes were ready to use. The ASC containing suspension was again strained as described above and centrifuged (5 min, 200 g). Afterward, the resulting cell pellet was incubated with erythrocyte-lysis buffer (155 mM ammonium chloride, 10 mM potassium hydrogen carbonate, 0.1 mM ethylenediaminetetraacetic acid) for 10 min at RT. Parallel to the next centrifugation step, cells were counted and seeded in MSCGMx (PeloBiotech, Planegg, Germany) with a cell density of 2 × 10^4^ per cm^2^.

### 4.3. Bioink Formulation and Model Generation

Gelatin methacryloyl (GelMA; GM2, Fraunhofer IGB, Stuttgart, Germany) was manufactured as previously published [72]. Claaßen et al. [73] determined the degree of functionalization as 85% by hydrogen-nuclear magnetic resonance spectroscopy.

For all following calculations, the total mass of the bioink was set as the reference value. Each cell type required a distinct bioink formulation whose composition for 1 mL bioink is summarized in Table 2.

*Manual Fabrication*

For both cellular bioinks, 100 µL bioink (cell suspension mixed in GelMA-LAP-solution) was filled into a round mold (8 mm diameter) with a 1 mL syringe (Braun Melsung, Melsung, Germany) and cooled down on ice for 10 s. Afterward, the constructs were immediately crosslinked under a UV light with 37 mW/cm^2^ for 180 s.

*Additive Fabrication*

The bioink was first transferred to the bioprinter cartridge (cellink, Boston, MA, USA) using a 1 mL syringe, which was further cooled down for 5 s from each side. After initial gelation, the cartridge was inserted with a nozzle into a temperature-controlled printhead, set to the desired temperature, and the bioprinting process in the BioX (cellink) was started. The exact bioprinting parameters can be taken from Figure 4. Additionally, the printing bed was tempered at 10 °C. After crosslinking with 37 mW/cm^2^ for 180 s, constructs were given directly into the cell culture medium.

*Culture Conditions*

ASC-containing constructs were cultured and differentiated for 14 days in DMEM supplemented with 10% fetal calf serum (Thermo Fisher Scientific, MA, USA), 1% P/S, 1 µM dexamethasone, 100 µM indomethacin, 500 µm isobutylmethylxanthine, and 1 µg/mL insulin to induce adipogenic differentiation. Media exchange with 750 µL was performed three times a week. Adipocyte-containing constructs were cultured in 750 µL AM1 supplemented with 1% P/S with twice media exchanges per week.

ASC- or MA-containing constructs were evaluated at different time points. ASCs were expected to differentiate for at least 14 days, while only a maintenance phase was targeted for MAs as mature cells.

### 4.4. Viscosity Determination

Viscosity determination was performed with a RheoStress 1 (Thermo Fisher Scientific, MA, USA) and a C35/1 cone (35 mm, 1° angle). Prior to measurement, the cone was rotated within the acellular GelMA (only mixed with PBS+ without cells) for 30 s, followed by a 30-s rest. The measurement started by lowering the cone onto the carrier plate, followed by rotating at a shear rate of 50 s^−1^ while the temperature of the carrier plate increased from 10 to 40 °C with 1 °C intervals.

### 4.5. Live-Dead Staining

For the live-dead staining, a staining solution with 8 µg/mL fluorescein diacetate (FDA; Sigma Aldrich, Taufkirchen, Germany), 20 µg/mL propidium iodide (PI; Sigma Aldrich), and 1 µg/mL Hoechst 33342 (Cell Signaling, MA, USA) in DMEM was prepared and incubated with the hydrogels at 37 °C for 15 min. Next, constructs were washed twice with PBS+ (each 15 min) and were imaged at RT with an Axio Observer microscope and an Axiocam 305 color camera using ZENblue software (all Carl Zeiss, Wetzlar, Germany).

### 4.6. Lactate Dehydrogenase Assay

LDH was analyzed in the cell culture supernatant via an LDH assay kit (Takara Bio, Shiga Prefecture, Japan). Determination was performed based on conditioned (24 h) cell culture supernatant. Therefore 50 µL cell culture supernatant and 50 µL LDH reagent (catalysator 1:45 dye) were mixed in a 96-well plate. This mixture was incubated for 30 min at RT in the dark and quantified via absorbance measurements at 490 nm with a reference wavelength set at 680 nm employing a spectrophotometer (Tecan Safire^2^, Männedorf, Switzerland).

### 4.7. Glycerol Measurement

Released glycerol was measured after 24 h of culture in the supernatant of the tissue constructs. The assay (Randox Laboratories, Crumlin, U.K.) was performed according to the manufacturer’s instructions. Briefly, samples were diluted 1:10 with buffer and quantified with a defined dilution series. Next, 100 µL assay reagent was added to the samples and the dilution series. Absorbance measurement took place after a 30 min incubation at 520 nm employing a spectrophotometer.

### 4.8. Staining of Intracellular Lipids

Intracellular lipids were stained with the lipophilic dye BodiPY 493/503 (Biomol). In addition, cell nuclei were stained with Hoechst 33342. The staining solution contained 1 µg/mL of each dye in PBS- (without divalent ions, Lonza Gampel-Bratsch, Switzerland). Hydrogels were incubated in 750 µL staining solution for 1 h at RT. Afterward, constructs were washed twice in PBS- for 15 min.

### 4.9. Anti-Perilipin A Staining

Hydrogels were fixed for 3 h in 4% paraformaldehyde (ROTI^®^Histofix, Carl Roth, Karlsruhe, Germany) and cut in half. Cells within the hydrogel were permeabilized for 30 min (0.1% Triton X-100 Sigma Aldrich T8787 in PBS-), blocked for 30 min, and washed three times for 15 min in washing buffer (0.1% Tween20 in PBS-). Staining was performed with 1:500 rabbit anti-human perilipin A antibody (Sigma Aldrich, P1998) for 1 h and afterward with 1:500 goat anti-rabbit IgG-Alexa488 antibody (ab60314, abcam, Cambridge, U.K.) for 30 min. Hydrogels were washed and stained with 4′,6-Diamidin-2-phenylindol (DAPI, Serva) for 10 min and washed again.

### 4.10. Hematoxylin-Eosin Staining

With 4% paraformaldehyde (Carl Roth, as all other reagents) fixed samples were embedded in paraffin, and sections of 5 µm were generated. Before hematoxylin-eosin staining, tissue sections were deparaffined according to a standard protocol and afterward stained according to a standard staining procedure (roticlear, 100%, 70%, 50% ethanol, hematoxylin, bluish, eosin, water, 70%, 100% ethanol, isopropanol). Sections were covered with FluoromountG (Life Technologies, Carlsbad, CA, USA) and a coverslip and analyzed by microscopy.

### 4.11. Image Quantification

Three images (live-dead staining) of each biological donor were counted manually without any software and displayed graphically for the semi-quantification of the presented images. The percentage of dead cells was determined by PI-stained cells, and the percentage of viable cells was determined by counting the FDA-stained cells, with the sum equal to 100%.

### 4.12. Statistics

If not stated otherwise, data were obtained from three different biological donors and a minimum of three technical replicates. All data were analyzed with GraphPad Prism 9.4.1 and are depicted as mean values ± standard deviation. Before the one-way analysis of variance (ANOVA), all data were analyzed for normal distribution and outliers. A *p*-value of ≤0.05 was considered statistically significant and indicated by a star throughout the manuscript.

## Figures and Tables

**Table 1:** Literature using GelMA for the construction of adipose tissue models.

Cell Type	Material	Bio-Printing	Research Findings	Reference
human ASC	10 *w/v*% GelMA	yes, EBB	Comparison of GelMA with a novel developed gelatin-based bioink, whereby GelMA serves as a positive control with high cell viability and adipogenic differentiation potential	[32]
human primary ASC	15 *w/v*% GelMA	no	GelMA-based hydrogels with adipogenic differentiated ASCs in a bioreactor, indicating a more stable differentiation in static culture	[33]
human primary ASC	5 or 10% GelMA and 5% GelMA + 4% Hyaluronic acid	no	Comparison of different GelMA variations, all suitable for adipogenic differentiation, composite shows more pronounced differentiation in some areas	[34]
human ASC	10 *w/v*% GelMA	yes, EBB	ASC spheroid encapsulated in GelMA and bioprinted, high viability and culture up to 14 days	[35]
immortal human ASC	5, 10, and 15 *w/v*% GelMA	no	Compared concentration-depended material characteristics of GelMA and cellular viability, which was highest in 5 *w/v*% GelMA	[40]
human primary mature adipocytes	5 wt% GelMA	no	Introduction of mature adipocytes to demonstrate the promising use of GelMA for this sensitive cell type	[36]

**Table 2:** Cell-containing GelMA composition.

Steps	Adipose-Derived Stem Cells	Mature Adipocytes
GelMA in PBS+	6.1 *w/v*% in 481.2 µL	6.1 *w/v*% in 361 µL
addition of unmodified gelatin	1.25 *w/v*%	
dissolving overnight at 37 °C and 5% CO_2_		
adding lithium-phenyl-2,4,6-trimethyl benzoyl-phosphinate (LAP)	3.8% with [3.22 mg/mL] in PBS+	
dissolving 1 h at 37 °C and 5% CO_2_		
adding cell suspension *	1 × 10^6^ cells per 481.2 µL PBS+	602 µL pure cell suspension

Reagents: PBS+ includes calcium and magnesium ions (HyClone, Fisher Scientific, Schwerte, Germany); LAP (Sigma Aldrich, Taufkirchen, Germany); bovine gelatin (bloom strength 300, GELITA Eberbach, Germany). * carried out in the dark, carefully stirred in with a cut pipette tip.

## Data Availability

The data presented in this study are available on request from the corresponding author.

## Supplementary Materials

The following are available online at https://www.mdpi.com/article/10.3390/gels8100611/s1, Table S1: semi-quantified values of cell viability; Figure S1 bioprinting evaluation of cell-containing GelMA: Bioprinting outcomes with insufficiently adjusted parameters. Left too low pressure and temperature, middle optimized parameters, right too high pressure. The phenomenon can be counteracted by adjusting the parameters in the scala below.
