# Supplementary material for: Bioprinting of 3D Adipose Tissue Models Using a GelMA-Bioink with Human Mature Adipocytes or Human Adipose-Derived Stem Cells
Source: Gels. 2022 Sep 25;8(10):611. doi: 10.3390/gels8100611 (PMC9601941; doi:10.3390/gels8100611)
Supplement: Supplementary file 1 [file gels-08-00611-s001.zip › Figure S1.pdf]

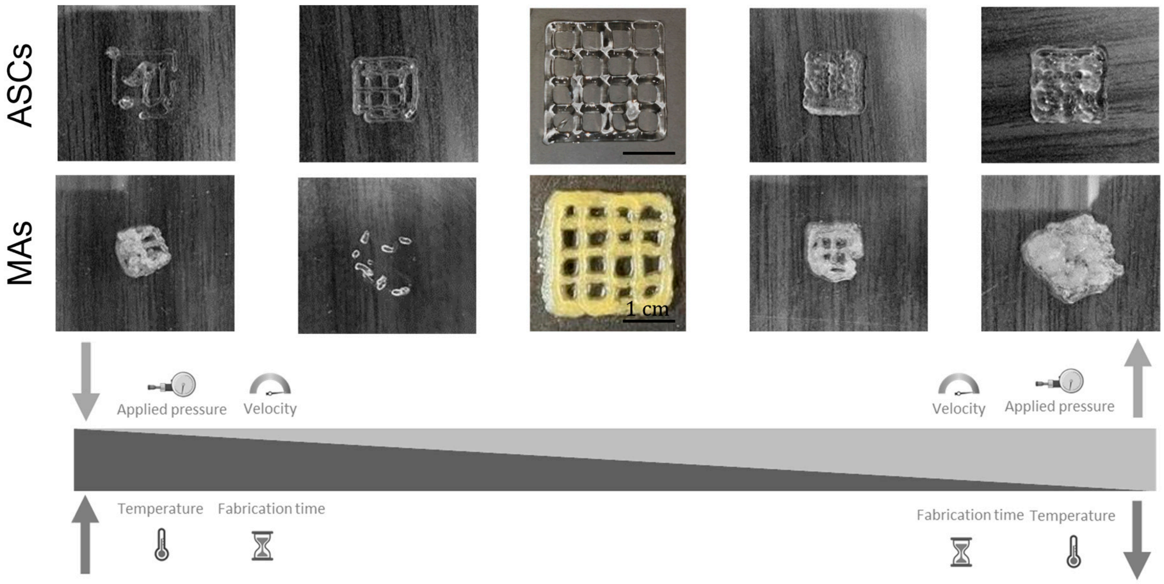

Supplementary Figure S1 bioprinting evaluation of cell-containing GelMA: Bioprinting outcomes with insufficiently adjusted parameters. Left too low pressure and temperature, middle optimized parameters, right too high pressure. The phenomenon can be counteracted by adjusting the parameters in the scala below.
