# Supplementary material for: Bioprinting of 3D Adipose Tissue Models Using a GelMA-Bioink with Human Mature Adipocytes or Human Adipose-Derived Stem Cells
Source: Gels. 2022 Sep 25;8(10):611. doi: 10.3390/gels8100611 (PMC9601941; doi:10.3390/gels8100611)
Supplement: Supplementary file 1 [file gels-08-00611-s001.zip › Table S1.pdf]

Table S1: semi-quantified values of cell viability

	condition	viable cells [%]	dead cells [%]	SD [%]
ASC in GelMA	day 1 manual	90.1	9.9	3.2
	day 1 additive	88.8	11.2	5.6
	day 15 manual	93.2	6.8	4.2
	day 15 additive	93.6	6.4	4.0
AC in GelMA	day 1 manual	85.1	14.9	6.2
	day 1 additive	84.0	16.0	7.6
	day 8 manual	86.2	13.8	8.8
	day 8 additive	87.8	12.2	10.2
explanted lobule	day 1	88.0	12.0	2.6
	day 8	87.3	12.7	7.9
